# Solving a Physiological Conundrum: Veno-Venous Extracorporeal Membrane Oxygenation (VV-ECMO) as a Bridge Strategy in Severe Acute Respiratory Distress Syndrome (ARDS) Complicated by High-Output Bronchopleural Fistula

**DOI:** 10.7759/cureus.116604

**Published:** 2026-09-20

**Authors:** Inês L Freitas, Joana S Braga, Ana R Branco, Francisco Teixeira da Silva, José Miguel Sá

**Affiliations:** 1 Intensive Care Department, Unidade Local de Saúde do Alto Minho, Viana do Castelo, PRT

**Keywords:** ards, bronchopleural fistula, hydropneumothorax, pulmonary empyema, vv-ecmo

## Abstract

Severe community-acquired pneumonia (CAP) may lead to complications such as septic shock, acute respiratory distress syndrome (ARDS), empyema, hydropneumothorax, and bronchopleural fistula, creating major therapeutic challenges. In patients with concomitant ARDS and a high-output bronchopleural fistula, the need for lung-protective ventilation may conflict with the reduction in airway pressures required to promote fistula closure. Veno-venous extracorporeal membrane oxygenation (VV-ECMO) may provide a bridge strategy by allowing ultraprotective ventilation while source control and antimicrobial therapy are pursued. We report the case of a 56-year-old man admitted with septic shock secondary to CAP complicated by empyema and hydropneumothorax. On admission, the patient met criteria for severe ARDS, with a PaO₂/FiO₂ ratio of 87 despite a positive end-expiratory pressure (PEEP) of 10 cmH₂O, and refractory hypoxemic and hypercapnic respiratory failure despite prone positioning. A persistent high-output bronchopleural fistula, associated with a continuous air leak of approximately 3 L/min, further compromised ventilatory management. Due to refractory respiratory failure, VV-ECMO was initiated within 24 hours of admission. During hospitalization, pleural cultures identified *Prevotella bivia*, followed later by *Acinetobacter baumannii*, and antimicrobial therapy was subsequently adjusted according to microbiological susceptibility and clinical response. VV-ECMO support enabled ultraprotective ventilation and reduced airflow through the fistulous tract, which was associated with a progressive reduction in fistula output and respiratory and structural recovery. This case highlights the physiological conflict between ARDS ventilation strategies and bronchopleural fistula management. VV-ECMO may provide a useful bridge strategy to facilitate ultraprotective ventilation in carefully selected patients with severe respiratory failure complicated by a high-output air leak and pleural infection, while definitive pleural source control and appropriate antimicrobial therapy are pursued.

## Introduction

Severe community-acquired pneumonia (CAP) remains a major cause of septic shock and acute respiratory distress syndrome (ARDS), particularly in patients with risk factors such as chronic alcohol abuse [1]. In a subset of patients, pulmonary infection progresses to complicated pleuropulmonary disease, including empyema, hydropneumothorax, and bronchopleural fistula, conditions associated with substantial morbidity and mortality [2-4].

Bronchopleural fistula is an uncommon but severe complication of necrotizing pulmonary infection and pleural disease. The presence of a persistent air leak significantly impairs effective ventilation and creates a major therapeutic dilemma in patients with concomitant ARDS. Lung-protective ventilation strategies in ARDS generally require appropriate positive end-expiratory pressure (PEEP) and airway pressures to maintain alveolar recruitment and gas exchange; however, excessive airway pressures may perpetuate and increase airflow through the fistulous tract, worsening the air leak and potentially delaying fistula closure [5,6]. Thus, the management of severe respiratory failure may directly conflict with the physiological conditions required for pulmonary healing.

Veno-venous extracorporeal membrane oxygenation (VV-ECMO) has emerged as a rescue therapy for refractory ARDS. Beyond providing gas exchange support, VV-ECMO enables ultraprotective ventilation with reduced tidal volumes and airway pressures, potentially minimizing airflow through bronchopleural fistulas and creating conditions favorable for tissue healing while definitive treatment of the underlying infection is pursued [5-9].

We report a case of septic shock secondary to severe CAP complicated by empyema, hydropneumothorax, and a high-output bronchopleural fistula resulting in refractory ARDS requiring VV-ECMO support. This case illustrates the potential role of VV-ECMO as a bridge strategy allowing simultaneous management of severe respiratory failure and bronchopleural fistula while source control, pleural drainage, and prolonged antimicrobial therapy are continued.

## Case presentation

A 56-year-old man with a history of chronic alcohol abuse was admitted to the emergency department after being found at home in a comatose state. On presentation, he exhibited severe respiratory distress with tachypnea and hypoxemia despite supplemental oxygen, tachycardia, mottled skin, and signs of peripheral hypoperfusion (Table 1).

**Table 1:** Clinical findings at admission

Parameter	Reported value	Reference range
Respiratory rate	40/min	12-18/min at rest
Oxygen saturation	86%	94-96%
Heart rate	150-160 bpm	60-100 bpm at rest

Arterial blood gas analysis demonstrated metabolic acidosis with elevated lactate, and laboratory studies revealed marked leukocytosis and elevated inflammatory markers (Table 2).

**Table 2:** Laboratory results at the emergency department

Parameter	Reported value	Reference range
Lactate	6.4 mmol/L	<2 mmol/L
White blood cell count	39,710/µL	4,500-10,000/µL
C-reactive protein	42 mg/dL	<1 mg/dL

Thoracic computed tomography demonstrated a large left pleural collection containing air foci suggestive of empyema, associated with ipsilateral atelectatic collapse and contralateral mediastinal shift. Multifocal right lung consolidations and ground-glass opacities were consistent with severe pneumonia (Figure 1).

**Figure 1:**
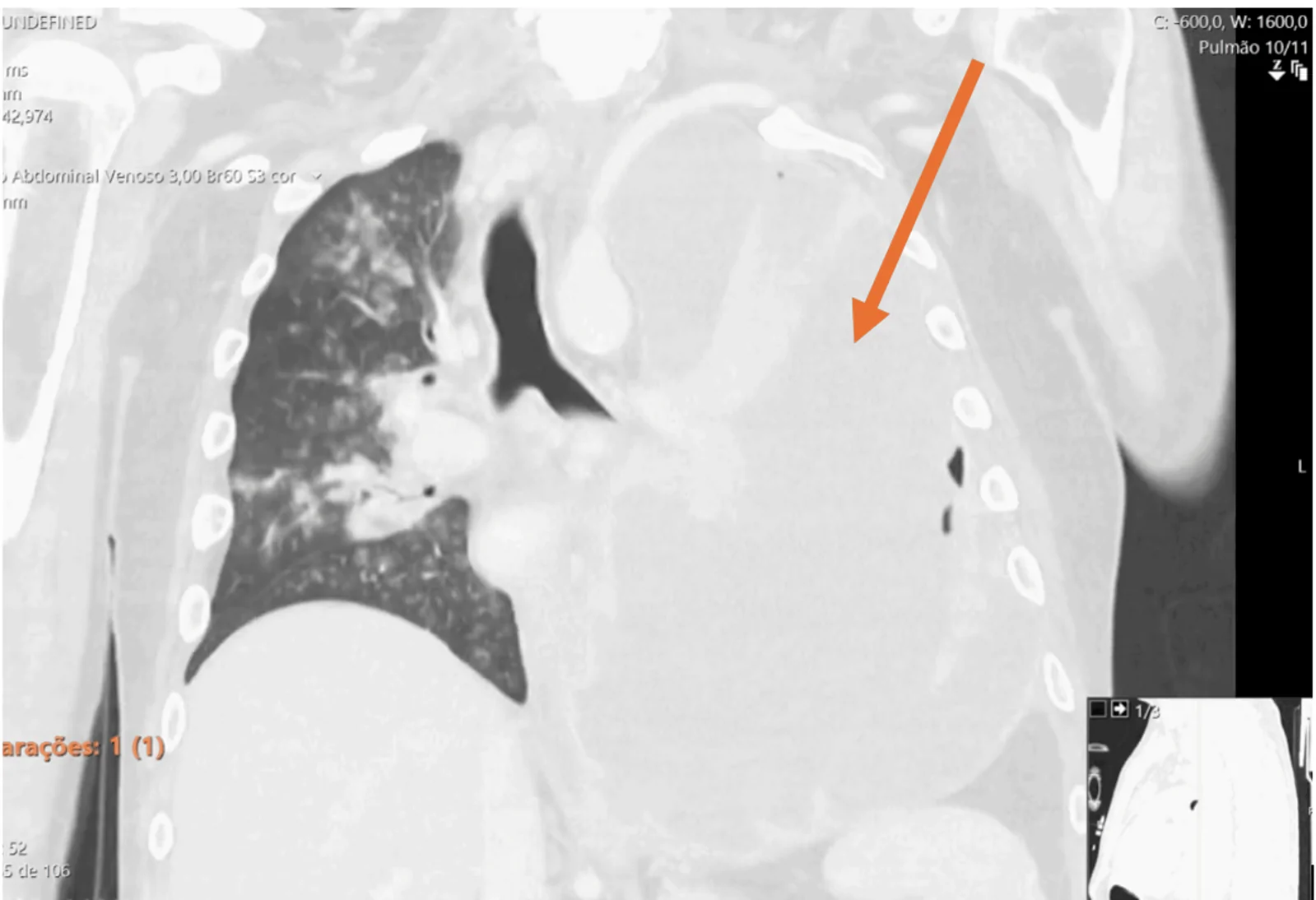
Thoracoabdominal computed tomography showing left pulmonary empyema (arrow), contralateral mediastinal shift, and right lung pneumonia (coronal view)

The patient was diagnosed with septic shock secondary to severe CAP complicated by pleural infection and multiorgan dysfunction. Initial severity assessment showed a Sequential Organ Failure Assessment (SOFA) score of 11 and a Simplified Acute Physiology Score II (SAPS II) score of 54 [10,11]. Hemodynamic stabilization required a norepinephrine infusion of up to 0.57 mcg/kg/min and vasopressin. Empiric antibiotic therapy with ceftriaxone and azithromycin was initiated, followed by admission to the intensive care unit (ICU), endotracheal intubation, and placement of a left chest tube with drainage of purulent pleural fluid compatible with empyema.

Despite lung-protective mechanical ventilation, the patient developed severe ARDS with refractory hypoxemia and acidemia associated with hypercapnia (pH 7.22, PaCO2 58 mmHg, HCO3- 23.7 mmol/L). The PaO₂/FiO₂ ratio was 87 despite a PEEP of 10 cmH₂O and optimization of ventilatory parameters, neuromuscular blockade, and prone positioning. Simultaneously, a persistent high-output air leak of approximately 3 L/min through the chest drainage system, detected by a continuous digital monitoring system, strongly suggested a bronchopleural fistula, significantly impairing effective ventilation and preventing adequate protective ventilatory strategies.

Given refractory respiratory failure and the inability to simultaneously maintain adequate gas exchange while minimizing airflow through the fistula, the patient was cannulated for VV-ECMO at our institution by the ECMO retrieval team within 24 hours of admission and subsequently transferred to a tertiary ECMO center.

During VV-ECMO support, ultraprotective ventilation was implemented with reduced tidal volumes and airway pressures. Follow-up imaging demonstrated persistent pneumonia and loculated left hydropneumothorax despite adequate chest tube positioning. Microbiological studies from tracheal aspirates and pleural fluid cultures initially failed to isolate a causative organism. Given gradual clinical improvement, antibiotic therapy was de-escalated to amoxicillin/clavulanate. Subsequent pleural fluid cultures identified *Prevotella bivia*.

Progressive improvement of the air leak and pulmonary mechanics allowed successful VV-ECMO decannulation after 12 days.

Upon return to the referring ICU, persistent infection and complications remained a management challenge (Figure 2).

**Figure 2:**
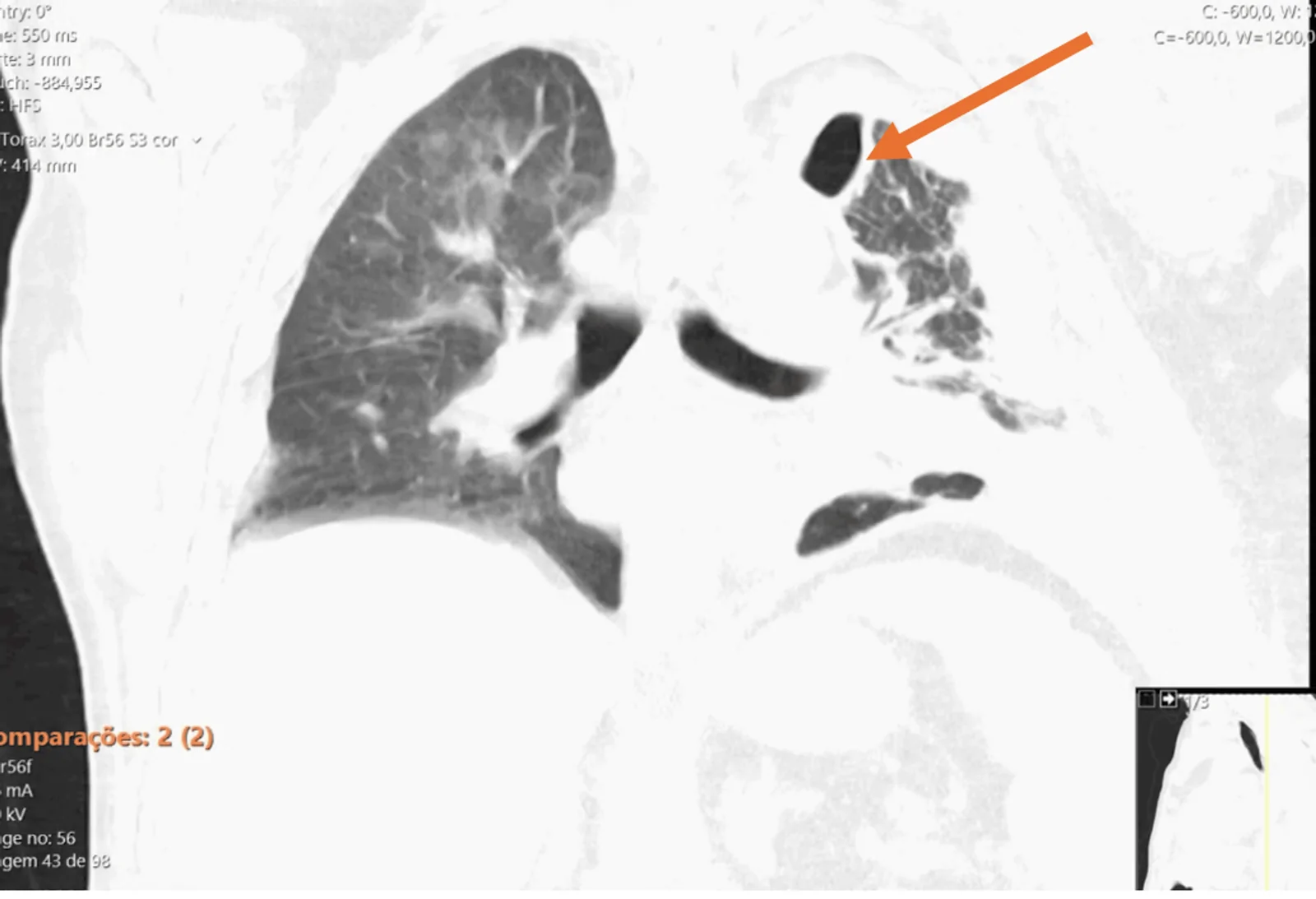
Thoracic computed tomography after 17 days of hospitalization showing persistent infection and pneumothorax (arrow, coronal view)

Additional pleural fluid cultures later isolated *Acinetobacter baumannii*, prompting targeted therapy with sulfamethoxazole/trimethoprim according to the isolate’s susceptibility profile. Given incomplete source control and the complexity of pleural infection, prolonged antibiotic therapy was maintained for a total duration of six weeks based on the clinical, microbiological, and radiological course.

Ventilatory weaning was achieved without the need for tracheostomy, with successful extubation after 14 days of invasive mechanical ventilation. The patient progressively improved, was transferred to the medical ward with domiciliary noninvasive ventilation after a total ICU stay of 21 days, and subsequently to a rehabilitation unit, where he completed antibiotic therapy with favorable clinical and radiological progress.

## Discussion

This case illustrates the major physiological challenge posed by the coexistence of severe ARDS and a high-output bronchopleural fistula complicating pleuropulmonary infection [12].

In this patient, the diagnosis of severe ARDS was supported not only by the severity of hypoxemia, with a PaO₂/FiO₂ ratio of 87 despite a PEEP of 10 cmH₂O, but also by the presence of substantial pulmonary parenchymal involvement. Thoracic CT demonstrated multifocal right lung consolidations and ground-glass opacities consistent with severe pneumonia, in addition to the left-sided empyema, atelectatic collapse, and hydropneumothorax. Thus, the bilateral pulmonary abnormalities and refractory hypoxemia were not considered to be explained predominantly by the pleural collection, lung collapse, or empyema alone, supporting the diagnosis of severe ARDS in the context of severe pleuropulmonary infection.

The central difficulty in this scenario lies in the conflicting ventilatory goals. ARDS management relies on lung-protective ventilation with adequate positive airway pressure to maintain alveolar recruitment and oxygenation. However, in the presence of a bronchopleural fistula, increased airway pressures increase airflow through the fistulous tract, perpetuating the air leak, impairing effective ventilation, and delaying fistula closure [5]. In this patient, the persistent air leak of approximately 3 L/min rendered conventional protective ventilation ineffective and contributed to refractory hypoxemic and hypercapnic respiratory failure despite prone positioning.

VV-ECMO provided a means of addressing this physiological conflict by allowing ultraprotective ventilation while maintaining adequate extracorporeal gas exchange. Although detailed ventilatory mechanics were not systematically recorded before VV-ECMO initiation, preventing objective quantification of the reduction in airway pressures achieved after extracorporeal support, the established “lung rest” strategy provided by VV-ECMO likely reduced airflow through the fistula while maintaining adequate oxygenation and carbon dioxide clearance [5-8]. The progressive reduction in air leak during VV-ECMO support suggests that minimizing ventilator-induced fistula flow likely facilitated structural healing.

An additional challenge was the coexistence of empyema and hydropneumothorax. The hydropneumothorax was consistent with the coexistence of infected pleural fluid and ongoing pleural air leakage in the setting of a bronchopleural fistula, illustrating the close interaction between pleural infection and structural pulmonary disruption [4]. Effective source control was therefore fundamental but difficult to achieve due to loculated pleural collections and prolonged pleural space infection, requiring several drainage procedures to achieve progressive radiological improvement [9]. In view of the favorable clinical and radiological evolution with pleural drainage, antimicrobial therapy, and VV-ECMO-supported ultraprotective ventilation, further invasive strategies, such as video-assisted thoracoscopic surgery or conventional thoracic surgery, were not ultimately required.

The microbiological evolution further reflects the complexity of severe pleuropulmonary infection. *Prevotella bivia*, an anaerobic organism associated with aspiration-related infection, was identified in initial pleural cultures in a patient with alcoholism, a recognized risk factor for aspiration [13]. Later isolation of *Acinetobacter baumannii* may represent secondary infection occurring in the context of prolonged hospitalization, structural lung damage, and impaired host defenses [14-16].

Overall, this report supports the role of VV-ECMO as a bridge strategy in selected patients with severe ARDS complicated by a bronchopleural fistula. Early initiation of VV-ECMO may permit ultraprotective ventilation while definitive pleural source control and treatment of the underlying infection are pursued, potentially facilitating conditions favorable for fistula closure when conventional ventilation may be physiologically incompatible with recovery (Figure 3).

**Figure 3:**
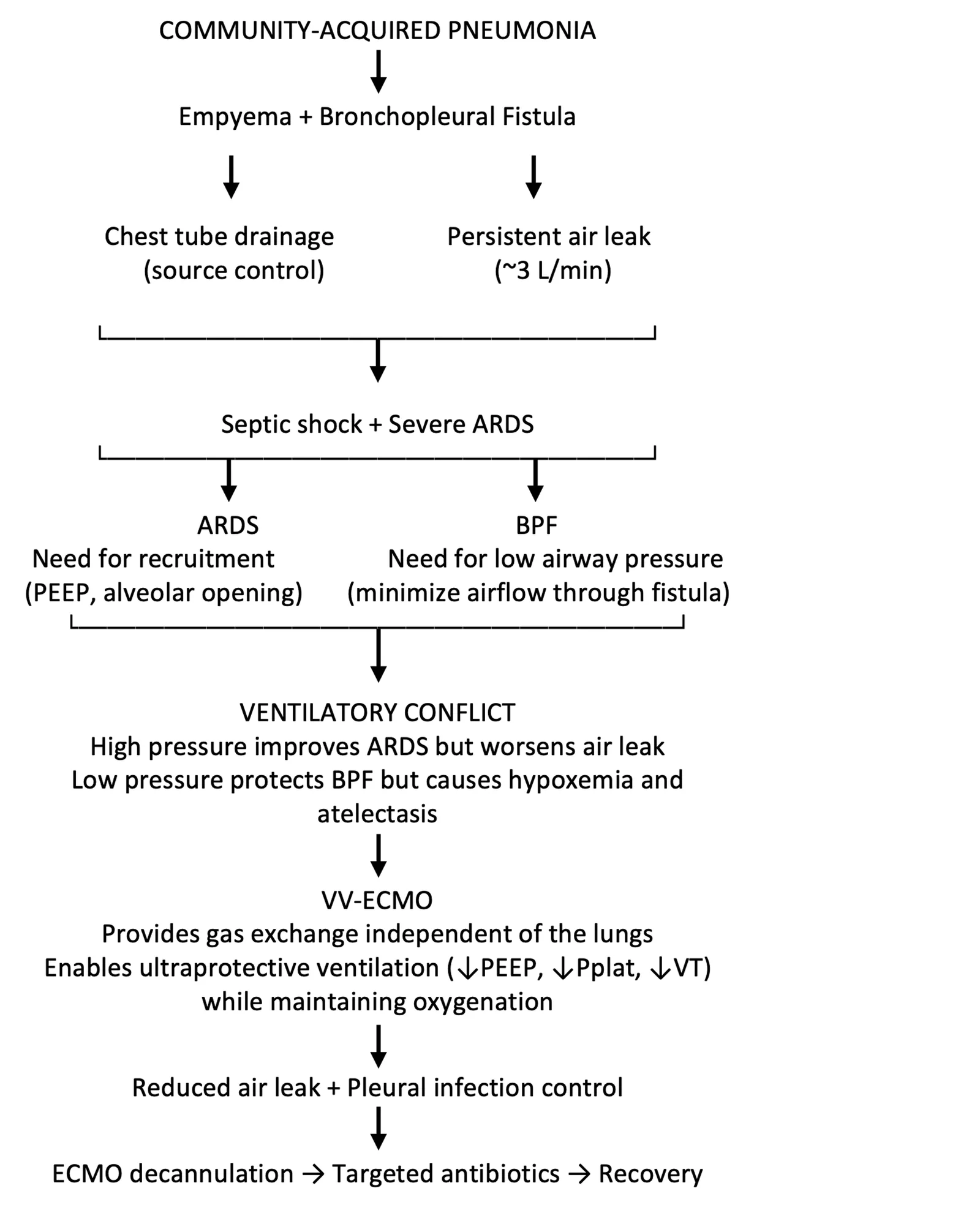
Conceptual summary of the clinical course and rationale for VV-ECMO ARDS, acute respiratory distress syndrome; BPF, bronchopleural fistula; PEEP, positive end-expiratory pressure; Pplat, plateau pressure; VT, tidal volume; VV-ECMO, veno-venous extracorporeal membrane oxygenation

## Conclusions

This case emphasizes the major therapeutic challenge created by the coexistence of severe ARDS, empyema with hydropneumothorax, and a high-output bronchopleural fistula. In this setting, the physiological requirements of lung-protective ventilation may directly conflict with the conditions necessary for fistula closure and pulmonary healing. VV-ECMO helped address this conflict by allowing ultraprotective ventilation and facilitating a reduction in airflow through the fistulous tract. This approach was associated with progressive fistula closure and structural lung recovery, while definitive treatment of the underlying pleuropulmonary infection was pursued. Successful management additionally depended on aggressive pleural source control, prolonged antimicrobial therapy, and multidisciplinary critical care management. Early recognition of these interacting pathophysiological mechanisms may improve outcomes in similarly complex cases. VV-ECMO may assist in transforming a physiologically incompatible ventilatory scenario into a potentially reversible condition in selected patients in whom conventional ventilation cannot adequately balance gas exchange requirements with the need to limit fistula flow.

## References

[1] Reyes LF, Conway Morris A, Serrano-Mayorga C, Derde LPG, Dickson RP, Martin-Loeches I (2025). Community-acquired pneumonia. Lancet.

[2] Dequin PF, Torres A, Bassetti M (2026). Severe community-acquired pneumonia: current concepts and controversies. Intensive Care Med.

[3] Elsheikh A, Bhatnagar M, Rahman NM (2023). Diagnosis and management of pleural infection. Breathe (Sheff).

[4] Papazachariou A, Papadokostaki E, Kypraiou D (2023). Hydropneumothorax as a complication of necrotizing pneumonia. Germs.

[5] Grotberg JC, Hyzy RC, De Cardenas J, Co IN (2021). Bronchopleural fistula in the mechanically ventilated patient: a concise review. Crit Care Med.

[6] Grasselli G, Calfee CS, Camporota L (2023). ESICM guidelines on acute respiratory distress syndrome: definition, phenotyping and respiratory support strategies. Intensive Care Med.

[7] Garlick J, Maxson T, Imamura M, Green J, Prodhan P (2013). Differential lung ventilation and venovenous extracorporeal membrane oxygenation for traumatic bronchopleural fistula. Ann Thorac Surg.

[8] Daoud O, Augustin P, Mordant P (2011). Extracorporeal membrane oxygenation in 5 patients with bronchial fistula with severe acute lung injury. Ann Thorac Surg.

[9] Roberts ME, Rahman NM, Maskell NA (2023). British Thoracic Society Guideline for pleural disease. Thorax.

[10] Vincent JL, Moreno R, Takala J (1996). The SOFA (Sepsis-related Organ Failure Assessment) score to describe organ dysfunction/failure. On behalf of the Working Group on Sepsis-Related Problems of the European Society of Intensive Care Medicine. Intensive Care Med.

[11] Le Gall J, Lemeshow S, Saulnier F (1993). A new Simplified Acute Physiology Score (SAPS II) based on a European/North American multicenter study. J Am Med Assoc.

[12] Matthay MA, Arabi Y, Arroliga AC (2024). A new global definition of acute respiratory distress syndrome. Am J Respir Crit Care Med.

[13] Fujita K, Takata I, Sugiyama H, Suematsu H, Yamagishi Y, Mikamo H (2019). Antimicrobial susceptibilities of clinical isolates of the anaerobic bacteria which can cause aspiration pneumonia. Anaerobe.

[14] Abeyrathna D, Wijekoon L, Kumarasinghe C, Weerawansa P, Siribaddana S (2025). Acinetobacter baumannii causing complex parapneumonic effusion in a community-living immunocompetent host: a case report. Cureus.

[15] Shi J, Cheng J, Liu S, Zhu Y, Zhu M (2024). Acinetobacter baumannii: an evolving and cunning opponent. Front Microbiol.

[16] Ren X, Palmer LD (2023). Acinetobacter metabolism in infection and antimicrobial resistance. Infect Immun.

